# Non‐Rigid Band Structure in Mg_2_Ge for Improved Thermoelectric Performance

**DOI:** 10.1002/advs.202000070

**Published:** 2020-04-30

**Authors:** Hasbuna Kamila, Aryan Sankhla, Mohammad Yasseri, Eckhard Mueller, Johannes de Boor

**Affiliations:** ^1^ Institute of Materials Research German Aerospace Center (DLR) Cologne 51147 Germany; ^2^ Institute of Inorganic and Analytical Chemistry Justus Liebig University Giessen Giessen 35392 Germany

**Keywords:** Mg_2_Ge, non‐rigid band structures, thermoelectric performance, thermoelectrics, valence band model

## Abstract

Magnesium silicide and its solid solutions are among the most attractive materials for thermoelectric generators in the temperature range of 500–800 K. However, while n‐type Mg_2_(Si,Ge,Sn) materials show excellent thermoelectric performance, the corresponding p‐type solid solutions are still inferior, mainly due to less favorable properties of the valence bands compared to the conduction bands. Here, Li doped Mg_2_Ge with a thermoelectric figure of merit zT of 0.5 at 700 K is reported, which is four times higher than that of p‐type Mg_2_Si and double than that of p‐type Mg_2_Sn. The reason for the excellent properties is an unusual temperature dependence of Seebeck coefficient and electrical conductivity compared to a standard highly doped semiconductor. The properties cannot be captured assuming a rigid band structure but well reproduced assuming two parabolic valence bands with a strong temperature dependent interband separation. According to the analysis, the difference in energy between the two bands decrease with temperature, leading to a band convergence at around 650 K and finally to an inversion of the band positions. The finding of a combination of a light and a heavy band that are non‐rigid with temperature can pave the way for further optimization of p‐type Mg_2_(Si,Ge,Sn).

## Graphical abstract

Sketch of the strongly temperature dependent band structure in Li‐doped Mg_2_Ge. With temperature the interband separation (Delta E) decreases leading to a convergence of the valence bands (heavy hole+light hole (HH+LH) and split‐off (SO)) and extraordinary thermoelectric properties at high temperatures. The dashed lines indicate the chemical potentials of the individual samples.

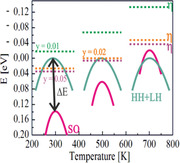

## 1 Introduction

Thermoelectric (TE) power generators are highly promising energy alternatives as they offer heat–electricity conversion and vice versa for a wide range of applications such as space applications, automotive, and refrigerators.^[^ ^1^ ^]^ Thermoelectric devices are highly reliable, compact, and the efficiency scales only weakly with size. The device efficiency depends monotonously on the dimensionless figure of merit *zT* = $\frac{S^{2}\sigma }{\kappa }$ *T*, where *S* is the Seebeck coefficient, *σ* is the electrical conductivity, *κ* is the thermal conductivity, and *T* is the temperature. A high *S*, *σ*, and low *κ* of the employed thermoelectric materials are required to achieve good thermoelectric properties.

In recent years, there has been progress in optimizing thermoelectric properties of p‐type Mg_2_X (X = Si, Ge, and Sn).^[^ ^2^ ^]^ However, the thermoelectric properties are still inferior in comparison with n‐type Mg_2_(Si, Sn, Ge).^[^ ^3^ ^]^ For further development of thermoelectric generators based on Mg_2_X, both good p‐and n‐type materials are highly desired.

As basic strategy, optimizing carrier concentration through doping can be employed to enhance thermoelectric properties.^[^ ^2b,c^ ^]^ Based on the expression *zT* scales with *S* ^2^, in order to achieve high *zT*, improving the Seebeck coefficient is more efficient than enhancing electrical conductivity. For degenerate semiconductors, it is well recognized that a large density of states effective mass ($m_{D}^{*}$) is beneficial for high *S* for a given carrier concentration. Large $m_{D}^{*}$ can be achieved either by high valley degeneracy (*N*_V_) and large single‐band effective mass (*m*_b_) since $m_{D}^{*}={N_{v}}^{2/3}\;m_{b}$.^[^ ^4^ ^]^ However, heavy *m*_b_ will give a low carrier mobility ($\mu \approx \frac{1}{{m_{b}}^{2.5}}$) and consequently reduces the electrical conductivity (*σ* = *neμ*). For an improvement of the thermoelectric performance beyond the basic carrier concentration optimization, advanced concepts need to be applied. These include band structure engineering (band flattening,^[^ ^5^ ^]^ band convergence,^[^ ^6^ ^]^ resonant level,^[^ ^7^ ^]^ temperature dependent band positions^[^ ^8^ ^]^) as well as scattering engineering^[^ ^9^ ^]^ (energy filtering effect^[^ ^2^, ^10^ ^]^ and modulation doping^[^ ^11^ ^]^). In particular, band convergence where different bands converge through alloying or changing temperature leads to an increase of the density of states effective mass without degrading the mobility and consequently increases *S* ^2^ *σ*. Band convergence has been “applied” in Mg_2_(Si, Sn)^[^ ^6a^ ^]^ and PbTe_0.85_Se_0.15_.^[^ ^12^ ^]^ As example, by alloying PbTe with specific elements (Mn,^[^ ^13^ ^]^ Mg,^[^ ^14^ ^]^ Cd,^[^ ^15^ ^]^ and Sr^[^ ^16^ ^]^) the convergence of electronic bands can be manipulated to take place in the desired temperature range.

In this work, we have synthesized p‐type Mg_2_Ge via high energy ball milling using Li as a dopant. The results show that the observed Seebeck coefficient first increases with increasing temperature and decreases slowly at higher temperatures. This is atypical for highly doped semiconductors, which usually show a decrease with temperature after a pronounced maximum. We have also observed a pronounced increase in electrical conductivity at temperatures below the onset of bipolar conduction. The temperature dependent Seebeck coefficient and electrical conductivity has been analyzed using two parabolic valence bands (2PVB) model. The model assumes the heavy hole (HH) and light hole (LH) band as one effective heavy band of Mg_2_Ge and the split‐off (SO) band as second band. We assume that there is a temperature dependent interband separation (Δ*E*) between the HH + LH band and the SO band. The results reveal that our experimental data and the model are in good agreement only when considering a temperature dependent interband separation. The almost constant Seebeck coefficient and an increase in *σ* at high temperature lead to a superior power factor and enhance *zT*_max_ ≈ 0.5 at 700 K.

## 2 Results

### 2.1 Microstructure

**Figure 1:**
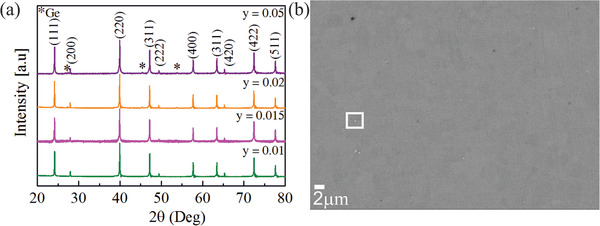
a) XRD patterns of Mg_2‐_ *_y_*Li*_y_*Ge with different Li contents (*y =* 0.01, 0.015, 0.02, and 0.05) and b) SEM–BSE images of Mg_1.98_Li_0.02_Ge region with white rectangle area corresponding to elemental Ge.

**Figure** **1**a shows XRD patterns of Li doped Mg_2_Ge samples. The main peaks can be indexed to an anti‐flourite cubic crystal structure with a space group Fm-3m (ICSD collection code #81735). Elemental Ge impurity peaks are observed for *y =* 0.02 and *y =* 0.05 and the intensity increases with increasing Li content. The observed elemental Ge could be due to unintended Mg loss, for example, from evaporation during the sintering process, or Mg lost to the jar walls during the ball milling,^[^ ^3^, ^23^ ^]^ or differences in the mechanical properties of Mg and Ge (ductile Mg and hard‐brittle Ge). However, why this would differ between the samples remains unclear. The lattice constant (*a*) is calculated by structural refinement (*a* = 6.392 Å) and it is in agreement with previous literature, 6.378–6.393 Å.^[^ ^24^ ^]^

Microstructure and phase purity of Mg_1.98_Li_0.02_Ge sample were observed using SEM. The grain size was estimated using ImageJ to be 4–5 µm, which is typical for samples of Mg_2_X synthesized using high energy ball milling.^[^ ^2^, ^3^, ^17^ ^]^ The white particles were observed in all examined samples (see Supporting Information). EDX point analysis indicates them to be elemental Ge in agreement with the XRD analysis.

### 2.2 Thermoelectric Properties

The thermoelectric transport data of p‐type Mg_2_Ge is listed in **Table** **1**. The Hall carrier concentration (*p*_H_), the density of states effective mass ($m_{D}^{*}$), and the Hall mobility (*μ*_H_) are calculated from the measured transport data using a single parabolic band (SPB) model.^[^ ^25^ ^]^

**Table 1:** Thermoelectric properties for p‐type Mg_2_Ge at room temperature calculated using a single parabolic band (SPB) model. Note that a SPB model does not describe the system very well, the numbers are therefore estimates only. More accurate parameters are provided in Table 2 in Section 3

Composition	*p*_H_ × 10^20^ [cm^−3^]	$m_{D}^{*}$ [m0]	*μ*_H_ [cm^2^ V^−1^ s^−1^]
Mg_1.99_Li_0.01_Ge	0.29	1.8	17
Mg_1.985_Li_0.015_Ge	0.61	2.1	35
Mg_1.98_Li_0.02_Ge	0.71	2.5	26
Mg_1.95_Li_0.05_Ge	0.93	2.5	30

**Figure 2:**
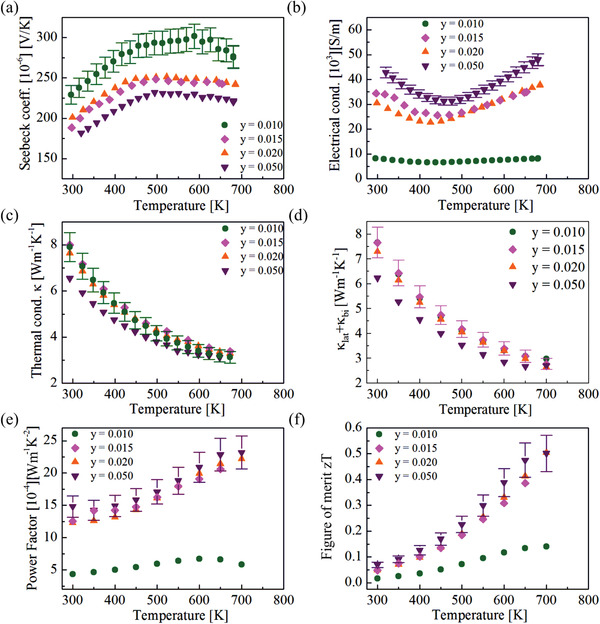
Temperature dependence of a) Seebeck coefficient, b) electrical conductivity, c) thermal conductivity, d) lattice + bipolar thermal conductivity, e) power factor, and f) figure of merit *zT* of Mg_2−y_Li_y_Ge with *y =* 0.01, 0.015, 0.02, and 0.05.

The thermoelectric properties of p‐type Mg_2_Ge are shown in **Figure** **2** and the transport data is taken from the cooling data (see Supporting Information). Figure 2a shows positive Seebeck coefficient values for all of the samples, which indicates p‐type conduction. The Seebeck coefficient for all samples shows first an increase with *T* followed by an almost constant value at higher temperatures. The starting temperature for this “plateau” shifts to higher temperature with higher Li concentration except for *y =* 0.015. *S* decreases with increasing Li concentration due to its inverse relation with carrier concentration. We also find a decrease in electrical conductivity for all samples till a minimum at 450 K followed by a relatively sharp increase at higher *T*. We have observed this unusual behavior, which is atypical for a highly doped semiconductor for all samples with the minimum shifting to higher *T* with increasing dopant or carrier concentration. The electrical conductivity increases with higher Li concentration due to an increase in the carrier concentration and the Hall mobility except for *y =* 0.015. The thermal conductivity and the lattice thermal conductivity show the same trend for all samples. The thermal conductivity of p‐type Mg_2_Ge has similar values as n‐type Mg_2_Si for a similar carrier concentration.^[^ ^26^ ^]^ The lattice thermal conductivity (including the bipolar contribution) is calculated using the Lorenz number (*L*) calculated for each band individually assuming a two parabolic valence band model, see Equation (8) in Section 3. An almost constant *T* dependent Seebeck and an increase in electrical conductivity lead to a strong increase of the PF with temperature. The PF increases dramatically for Li doped samples *y =* 0.01 and *y =* 0.015 from 0.6 to 2.0 mW^−1^ K^−2^ at 650 K. The highest PF is achieved for Mg_2−_ *_y_*Li*_y_*Ge with *y =* 0.05 (2.3 mW^−1^ K^−2^ at 700 K). The high PF leads to the highest figure of merit *zT* for *y =* 0.02 and *y =* 0.05 *zT*_max_ = 0.50 ± 0.07 at 700 K.

The SPB model is used to calculate $m_{D}^{*}$ and *μ*_H_ at room temperature under the assumption of a rigid band structure and that the $m_{D}^{*}$ is independent of carrier concentration.^[^ ^25^ ^]^ The reduced chemical potential (*η*) given by $\eta =\frac{E_{F}}{k_{B}T}$ is calculated using the relation with the Seebeck coefficient $S=\frac{k_{b}}{e}\;\left(\frac{2F_{1}\left(\eta \right)}{F_{0}\left(\eta \right)}-\eta \right)$, with the Fermi integral of order $\left(i\right)\;F_{i}=\int_{0}^{\infty }\frac{\in^{i}d\in }{1+\mathrm{Exp}[\in -\eta ]}$, *E*_F_ as Fermi energy and *k*_B_ as Boltzmann's constant. The true carrier concentration (*p*) is calculated from the Hall pre‐factor (*r*_H_) and the “measured” *p*_H_, $p_{H}=\frac{1}{R_{H}e}=\frac{p}{r_{H}},r_{H}=\frac{1.5F_{0.5}\left(\eta \right)\left(0.5\right)F_{-0.5}\left(\eta \right)}{F_{0}^{2}\left(\eta \right)}$. $m_{D}^{*}$ is calculated using the relation $p=4\pi {\left(\frac{2m_{D}^{*}k_{B}T}{h^{2}}\right)}^{1.5}\;F_{0.5}\left(\eta \right)$. *μ*_H_ at room temperature is calculated using a relation $\mu_{H}=\frac{\sigma }{p_{H}e}$. For the calculations, we have assumed a scattering parameter *λ* = 0 corresponding to the energy dependence of acoustic phonon scattering.^[^ ^25c^ ^]^ As results from the SPB calculation, the carrier concentration and the density of states effective mass increase with higher Li concentration. The highest Hall mobility is obtained for *y =* 0.015, while the other three Li doped samples exhibit an increase with higher Li content.

### 2.3 Modeling of the Thermoelectric Properties of p‐Mg_2_Ge

**Figure 3:**
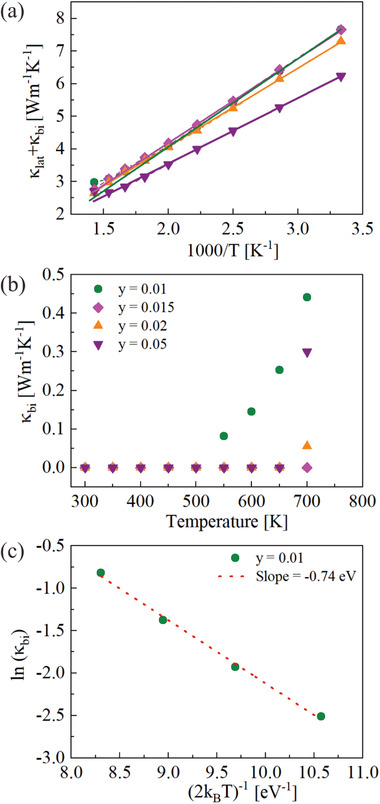
a) The difference of total thermal conductivity and electronic thermal conductivity as a function of temperature for p‐type Mg_2_Ge, the solid lines are linear fits. b) The bipolar contribution is calculated from the difference between the linear fitting and the *κ*_lat_ + *κ*_bi_. c) Plot of ln(*κ*_bi_) versus (*2k*_B_ *T*)^−1^ for Mg_1.99_Li_0.01_Ge to calculate the band gap.

Atypical transport properties (unusual *T*‐dependence of *S*(*T*), *σ*(*T*), and increasing mobility with increasing carrier concentration) were observed for Li doped Mg_2_Ge. While quantitatively different (see ref. [26]) the temperature dependence of Seebeck coefficient and electrical conductivity resemble on a first glance the behavior of doped semiconductors when minority carriers contribute significantly to the transport. This is not the case for Mg_2_Ge as can be inferred from the analysis of the thermal conductivity data. At high temperature the minority carriers generated by thermal excitation not only decrease *S*, but also increase the thermal conductivity due to bipolar diffusion. The bipolar thermal conductivity *κ*_bi_ can be obtained from the measured total thermal conductivity *κ*_tot_ using Equation (9) where *κ*_lat_ is estimated assuming *κ*_lat_ ∝ *T* ^−1^.^[^ ^27^ ^]^ The difference of total thermal conductivity and bipolar thermal conductivity as a function of *T* ^−1^ for p‐type Mg_2_Ge is shown in **Figure** **3**a and the extracted bipolar thermal conductivity *κ*_bi_ at high temperature in Figure 3b. It is clearly seen that the *κ*_bi_ is non‐negligible only at ≥550 K (for Li = 0.01) while the upturn in electrical conductivity is observed at 450 K (see Figure 3b). For the other samples, the difference in temperature where an increase in electrical conductivity and in the bipolar thermal conductivity becomes visible is similar or larger. From this, we conclude that the observed upturn in electrical conductivity of Li doped samples cannot be explained by thermal excitation of minority carriers.

In addition, the band gap *E*_G_ can be roughly estimated from the relationship in Equation (7).^[^ ^27^, ^28^ ^]^ Plotting ln(*κ*_bi_) versus (2*k*_B_ *T*)^−1^ for Mg_1.99_Li_0.01_Ge shows a good linear fit and yields *E*_G_ = 0.74 eV which fits with previous reports.^[^ ^29^ ^]^ Comparison of the experimental result for *κ*_bi_ with the predicted one allows to estimate the temperature dependence of the band gap, yielding *E*_*G*_ = 0.74 − 5.85 × 10^−4^ *T* see Figure S6, Supporting Information. While the numbers here are a rough estimate (due to the small magnitude of *κ*_bi_ ). This relatively large band gap supports the conclusion that the influence of the minority carriers is not the reason for the observed trends in the transport data.

As the observed *T* dependences of the TE properties (*S* and *σ*) are not comparable to those of degenerate semiconductors, it is unlikely that the thermoelectric properties of Li doped Mg_2_Ge can be modeled using a SPB model like in the case of p‐type Mg_2_(Si, Sn).^[^ ^23^ ^]^ They furthermore do not resemble to those of (highly) doped semiconductors with some influence of the minority carriers at high *T* (which is already beyond SPB), and cannot be explained by a one conduction band (CB) and one valence band (VB) model as we show in the Supporting Information. By modeling the temperature dependent transport data using a 1CB + 1VB model in two cases (one with a relatively large band gap at high temperatures, the second with a smaller band gap) we can show that the experimental data cannot be reproduced by a 1CB + 1VB model, see Figures S4 and S5, Supporting Information. The observed experimental data at high temperature is therefore not due to a minority carrier effect. We have also observed that the density of states effective mass is dependent on carrier concentration for Li doped samples; this is an indication for non‐parabolicity or a non‐rigid multiband structure.

In the following, we will try to reproduce the thermoelectric properties of Li doped Mg_2_Ge using a 2PVB model based on previous calculations of the band structure of Mg_2_Ge.^[^ ^30^ ^]^ As discussed in Supporting Information the consideration of a conduction band was not deemed necessary, as the band gap of Mg_2_Ge is relatively large and the influence of the minority carrier is minor and visible only at high temperatures (Figures S4–S6, Supporting Information). The valence band maxima is lying at the Γ point in the Brillouin zone (BZ) with three bands having different effective masses, which are labeled as heavy hole (HH), light hole (LH), and split‐off (SO) bands, respectively. The HH and LH bands maxima are degenerate while the SO band is at a different energy due to spin‐orbit coupling.^[^ ^30a^ ^]^ The relevant equations are^[^ ^25^, ^30^, ^31^ ^]^

(1) $$m_{D}^{*}={\left(m_{D}^{*},1^{1.5}+m_{D}^{*},2^{1.5}\right)}^{2/3}$$

(2) $${\mu_{H}}_{1,2}=\frac{4}{3}\frac{{F_{0}}^{2}\left(\eta_{1,2}\right)}{F_{0.5}\left(\eta_{1,2}\right)F_{-0.5}\left(\eta_{1,2}\right)}\frac{e\pi \hbar^{4}}{\sqrt{2}{\left(k_{b}T\right)}^{1.5}{E_{\mathrm{Def}}}^{2}{\left(m_{1,2}\right)}^{2.5}}C_{l}$$

(3) $$\sigma_{1,2}={\mu_{H}}_{1,2}\frac{p_{1,2}}{r_{H1,2}}\;e$$

(4) $$\sigma_{tot}=\sigma_{1}+\sigma_{2}$$

(5) $$S_{\mathrm{tot}}=\frac{S_{1}\sigma_{1}+S_{2}\sigma_{2}}{\sigma_{\mathrm{tot}}}$$

(6) $$R_{H}=\frac{R_{H1}\sigma_{1}^{2}+R_{H2}\sigma_{2}^{2}}{{\left(\sigma_{1}+\sigma_{2}\right)}^{2}}=\frac{p_{1}r_{H,1}e\mu_{1}^{2}+p_{2}r_{H,2}e\mu_{2}^{2}}{{\left(p_{1}e\mu_{1}+p_{2}e\mu_{2}\right)}^{2}}$$

(7) $$\kappa_{\mathrm{bi}}=\frac{{{\left(S_{1}-S_{2}\right)}^{2}}_{\sigma_{1}}{\;}_{\sigma_{2}}}{\left({}_{\sigma_{1}}{+}_{\sigma_{2}}\right)}T\approx A\exp\left(\frac{-E_{G}}{2k_{B}T}\right)$$

(8) $$L_{1,2}={\left(\frac{k_{b}}{e}\right)}^{2}\frac{3F_{0}\left(\eta_{1,2}\right)F_{2}\left(\eta_{1,2}\right)-4F_{1}^{2}\left(\eta_{1,2}\right)}{F_{0}{\left(\eta_{1,2}\right)}^{2}}$$

(9) $$\kappa =\kappa_{e1,2}+\kappa_{\mathrm{lat}}+\kappa_{\mathrm{bi}}=\left(L_{1}\;\sigma_{1}+L_{2}\sigma_{2}\right)T+\kappa_{\mathrm{lat}}+\kappa_{\mathrm{bi}}$$

Here, subscript 1 and 2 refer to the transport properties of carriers in the individual bands. The density of states effective mass is the total of the band masses (Equation (1)) and is taken from the Pisarenko plot (see Supporting Information) using the SPB model as we are the first to provide transport data for highly doped p‐type Mg_2_Ge. We furthermore assume acoustic phonon scattering (Equation (2)), corresponding to the scattering parameter *λ* = 0. The Hall mobility (*μ*_H_) is calculated using Equation (2), where *C*_l_ is an elastic constant of Mg_2_Ge (1.17 × 10^11^ Pa)^[^ ^32^ ^]^ and *E*_Def_ is the deformation potential which characterizes the interaction between holes and phonons (*E*_Def_ = 9 eV).^[^ ^23^ ^]^ We kept the deformation potential constant based on our previous calculation on p‐type Mg_2_(Si, Sn)^[^ ^23^ ^]^ and because no further information is available. The total electrical conductivity is calculated from each band using Equations (3) and (4). The total Seebeck coefficient is calculated from the individual band contributions; the band with higher electrical conductivity is more strongly weighted. As *S* usually decreases with the number of carriers, whereas conductivity increases (see Equation (3)), the total *S* will generally be closer to the smaller *S* of two bands.

As a first attempt, we have assumed the HH and the LH bands are degenerate and did not include the SO band. There are three unknown parameters as inputs for the first model ($m_{D}^{*},$ mass ratio ($\frac{m_{\mathrm{LH}}}{m_{\mathrm{HH}}}$) and *p*). We have systematically tried to tune the ratio of effective mass $\frac{m_{\mathrm{LH}}}{m_{\mathrm{HH}}}$ and adjust *p* roughly so the model fits with the *σ* or *S* experimental data at least at room temperature (see Figures S8 and S9, Supporting Information). The modeled data does not fit with the experimental results as in this case the electrical conductivity decreases with increasing *T* independent of mass ratio. This is also observed for the Seebeck coefficient data where *S* naturally increases with increasing temperature which is inconsistent with our experimental data.

**Figure 4:**
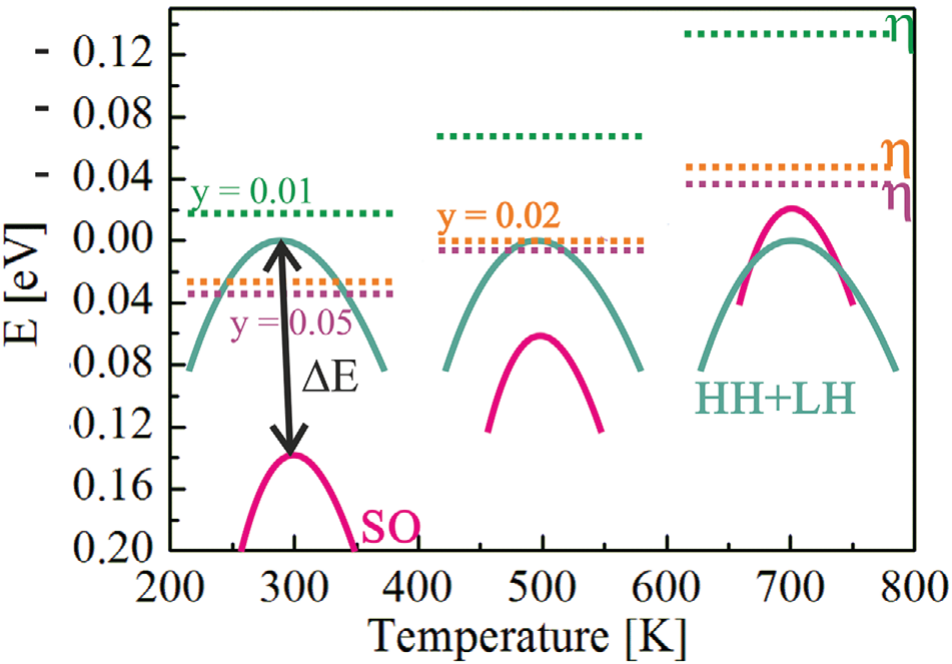
Schematic of the movement of the SO band (magenta) with respect to the HH + LH band (cyan) with temperature dependent interband separation (Δ*E*) for Mg_2−y_Li_y_Ge (*y =* 0.01, 0.02, and 0.05). The chemical potential (*η*; dashed lines) increases with higher Li concentrations and decreases as temperature increases.

In a second attempt, we have again assumed two bands with different curvatures. The LH and HH bands are considered as one effective band and the split‐off band as the second band. We did not attempt modeling of the properties using three individual valence bands because adding one more band introduces more adjustable parameters leading to an under‐defined model. This assumption is in agreement with band structure calculations of Mg_2_Ge, where the HH and LH are found to be degenerate.^[^ ^30^, ^33^ ^]^ The (HH + LH) band and the SO band are separated by an interband separation (Δ*E*) which was calculated to Δ*E*≅0.2 *eV* at 0 K.^[^ ^30a^ ^]^ The valence band structure of Ge and Mg_2_Ge are similar and for Ge, a temperature dependent Δ*E* was observed.^[^ ^34^ ^]^ Splitting between HH and LH has been observed for thin films of SiGe under strain^[^ ^35^ ^]^ which is not comparable to our case. Also, we found that the mass ratio for Mg_2_Ge $\frac{m_{\mathrm{HH}}}{m_{\mathrm{LH}}}\approx 6$ ^[^ ^34b^ ^]^ is similar with Ge^[^ ^36^ ^]^ which does not fit with our model, making the assumption of splitting of HH and LH implausible. Thus, we have assumed a band structure that is non‐rigid band with temperature with the HH and LH band as one effective band and the SO band as the lighter, second. We kept the effective mass of the SO band to be constant $\frac{m_{\mathrm{HH}+\mathrm{LH}}}{m_{\mathrm{SO}}}\approx 3.8;m_{\mathrm{SO}}=0.5\;m_{0,}\;m_{D}^{*}=2.1\;m_{0}$ which is similar with the case for Ge $\frac{m_{\mathrm{HH}}}{m_{\mathrm{SO}}}\approx 3.6$.^[^ ^36^ ^]^ We believe that the increase in electrical conductivity can be explained by a light band that moves up in energy with temperature like in the case of GaAs^[^ ^37^ ^]^ with the HH + LH band as the reference band. A strong temperature dependence of the interband separation Δ*E* is rare but has been observed in a few high performing thermoelectric materials.^[^ ^38^ ^]^ We have therefore chosen Δ*E* = (*A* + *B*/K*T) with *A* being negative and the linear form due to simplicity. The choice of Δ*E* = 0.26 at 0 K is similar to the value from DFT calculation of the band structure at 0 K.^[^ ^30a^ ^]^ The chemical potential is displayed for all samples (**Figure** **4**): it shifts down with higher Li concentrations and decreases as temperature increases.

The parameters that reproduce our experimental data reasonably well are listed in the **Table** **2**.

**Table 2:** Input parameters for the modeling of p‐type Mg_2_Ge, other (constant) parameters are: $E_{\mathrm{Def}}=9\;\mathrm{eV},m_{D}^{*}=2.1m_{0}$ and *C*_l_ = 1.17 × 10^11^ Pa

Composition	*m*_SO_ [m_0_]	*m*_HH + LH_ [m0]	*p* × 10^20^ [cm^−3^]	Δ*E* [eV]
Mg_1.99_Li_0.01_Ge	0.5	1.93	0.3	(−0.26 + 4 × 10^−4^/K*T)
Mg_1.985_Li_0.015_Ge	0.5	1.93	1.1	(−0.26 + 4 × 10^−4^/K*T)
Mg_1.98_Li_0.02_Ge	0.5	1.93	1.1	(−0.26 + 4 × 10^−4^/K*T)
Mg_1.95_Li_0.05_Ge	0.5	1.93	1.3	(−0.26 + 4 × 10^−4^/K*T)

**Figure 5:**
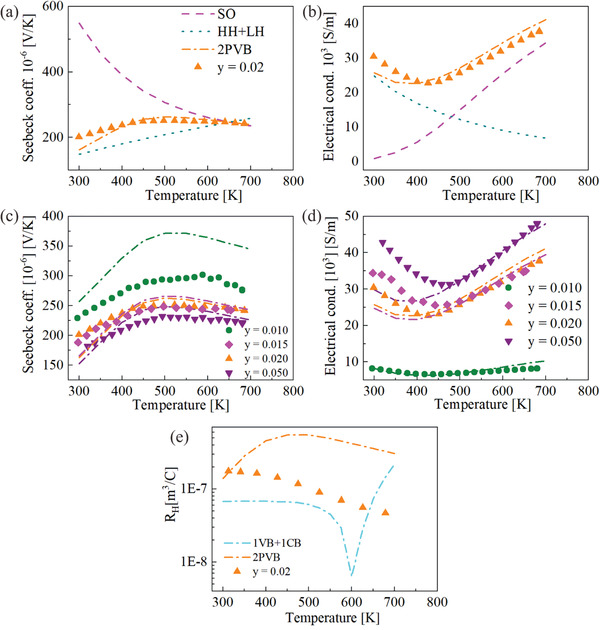
Temperature dependence of the measured a) Seebeck coefficient and b) electrical conductivity (symbols) and the contribution of the HH + LH band (dotted lines) and the SO band (dashed lines) are shown. The sum of both bands (dashed dotted lines) matches with experimental data. c,d) Exhibit the model and the experimental data for different Li concentrations. e) Temperature dependence of the measured Hall coefficient (*R*_H_) for Mg_1.98_Li_0.02_Ge together with the results from the 2PVB model (orange dashed dotted lines) and a 1VB + 1CB system (cyan dashed dotted lines) with a relatively large band gap at high *T* (*E*_G_ (*T*) = 0.57 − 1.8 × 10^−4^ *T*).

**Figure** **5** displays the modeled properties which qualitatively reproduce the experimental data. The plateau in *S*(*T*) and the upturn in electrical conductivity can be explained by the movement of the SO band: as temperature increases this leads to a decrease of Δ*E* and a convergence of the bands at 650 K. The Seebeck coefficient at room temperature is mainly governed by the HH + LH band. At room temperature, the HH + LH band contributes more to *σ* than the SO band thus *S* of 2PVB will be closer to *S* of the HH + LH band while at high temperature, the Seebeck coefficients of both bands converge. Figure 5b shows that the contribution to electrical transport majorly comes from the HH + LH band at low temperature while at high temperature the contribution to electrical transport from the SO band is dominant. The carriers in the lighter SO band have a much higher mobility and at higher temperatures, their fraction increases significantly while the fraction of the slower carrier decreases. Thus, even although both SO and HH + LH mobility decrease with temperature individually, the total conductivity increases with temperature, explaining the experimentally observed upturn.

We found the temperature dependent *S* and *σ* to be quite sensitive to Δ*E* (see Figures S10–S13, Supporting Information). The difference between the modeled and the experimental Seebeck coefficient at 300–500 K is getting smaller for lower value of Δ*E* in the Table 2 (see Figure S12, Supporting Information) however the temperature dependent Seebeck coefficient at high temperatures does then not resemble our experimental data. The fit between model and experimental data for *y =* 0.01 improves if we adjust the effective masses, however we do not have enough data to substantiate a carrier concentration dependent *m*_SO_^[^ ^39^ ^]^ (see Figure S15, Supporting Information). Since the SO band contributes mainly to the electrical conductivity, the effective mass of the SO band affects the temperature dependence of electrical conductivity. The lighter the mass of the SO band, the earlier (in temperature) and the sharper is the increase of electrical conductivity (Figure S16, Supporting Information).

Comparing the experimental result of the temperature dependent Hall coefficient with the prediction for the 2PVB system we do not find total agreement. The agreement is good at low temperatures, but the temperature dependence of *R*_H_ is different between 300 and 450 K. At higher temperatures, both the prediction and the experimental result show visible, but relatively weak temperature dependence. This is in agreement with the predicted behavior for a system with a convergence of the valence bands.^[^ ^40^ ^]^ Quantitatively the values agree within a factor of 10 over the whole temperature range. Possible reasons for the observed deviation are discussed in Supporting Information. For comparison, the results for a system with 1 CB and 1 VB are also shown (see Figure S7, Supporting Information). For this system, a change of sign of *R*_H_ is predicted (due to the higher mobility of the electrons) as well as much stronger temperature dependence. This is in clear disagreement with the experimental data.

In summary, the observed unusual thermoelectric properties can be explained by a two valence band model with different but constant effective masses and a temperature dependent interband separation.

## 3 Discussion

The atypical thermoelectric transport properties of Li doped Mg_2_Ge such as a nearly constant *S* at high *T* and a pronounced upturn in electrical conductivity cannot be explained by previously reported mechanisms such as energy filtering,^[^ ^2^, ^10^ ^]^ resonant levels,^[^ ^7^ ^]^ and modulation doping.^[^ ^11a^ ^]^ Energy filtering and modulation doping are ruled out because Ge precipitates are not evenly distributed at the grain boundaries as observed from the SEM images (see Figure 1). Another possibility could be the formation of secondary phases as GeLi and Li which could exist around our sintering temperature.^[^ ^24^ ^]^ In principle secondary phases like GeLi and Li can cause modulation doping, however the observed secondary phases are very inhomogeneously distributed and therefore unlikely to cause such phenomenon.^[^ ^41^ ^]^ The large *S* and the upturn in *σ* in Li doped Mg_2_Ge sample cannot be explained by resonant levels or resonant scattering effects like for Tl doped PbTe.^[^ ^42^ ^]^ First, the temperature dependence of the electrical conductivity shows a different trend compared to the electrical conductivity in the present study^[^ ^7^, ^43^ ^]^ and second in our case the electrical conductivity plays an important role in increasing the figure of merit while in the case of Tl doped PbTe the largely enhanced Seebeck coefficient was the main reason.

More or less constant values for the Seebeck coefficient at higher temperatures were also observed in other material systems such as Na doped PbTe‐PbS,^[^ ^41^ ^]^ PbTe_1‐_ *_x_*Se*_x_*,^[^ ^12^ ^]^ In doped GeTe,^[^ ^44^ ^]^ K doped PbTe,^[^ ^8^ ^]^ and CaZnAgSb Zintl phase.^[^ ^45^ ^]^ The measured temperature dependent data of *S* and *σ* can be explained by a temperature induced band order evolution similar to PbTe_1‐_ *_x_*Se*_x_*, Na doped PbTe‐PbS, and K doped PbTe.^[^ ^8^, ^12^, ^41^ ^]^ The movement of the light band with respect to the heavy band as temperature increases and the band convergence at high temperature are the causes for the good thermoelectric properties at high temperature in the previous cases while in our case, the light band being highest in energy is identified as the reason.

We have also observed that the carrier concentrations obtained from the Hall measurements are different from those of the 2PVB model especially for high Li concentrations (see Tables 1 and 2). This is presumably because the SO band is further off from *η* and the distance between *η* and the SO band is getting smaller with higher Li concentrations (see Figure 4), thus the deviation from SPB is larger for highly doped samples compared to low doped samples. Thus, the carrier concentration and Hall mobility obtained from SPB calculation do not have a strict physical meaning since they both are obtained from *R*_H_ under the (incorrect) assumption that a SPB model is applicable.

**Figure 6:**
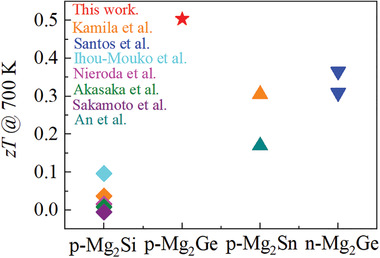
The experimental *zT* of p‐Mg_2_Si (♦),^[^ ^23^, ^46^ ^]^ p‐Mg_2_Ge (★this work), p‐Mg_2_Sn (▲),^[^ ^23^, ^47^ ^]^ and n‐type Mg_2_Ge (▼)^[^ ^48^ ^]^ at 700 K. P‐type Mg_2_Ge displays a higher *zT* than the other binaries p‐Mg_2_X and n‐type Mg_2_Ge.

**Figure** **6** shows the experimental *zT* of p‐type Mg_2_X and n‐type Mg_2_Ge at 700 K. The *zT* of p‐type Mg_2_Si is the lowest, mainly due to the experimental difficulties to obtain highly p‐doped samples; therefore minority carrier effects reduce the figure of merit at high temperature. Better properties are expected with increasing carrier concentration.^[^ ^23^ ^]^ However, our results for p‐Mg_2_Ge are also superior to those for Mg_2_Sn, where *zT* can experimentally optimized with respect to carrier concentration. Given that the thermal conductivities are somewhat similar, the main improvement comes from the electronic transport. We also note that *zT*_max_ of Li doped Mg_2_Ge is comparable to the best p‐type solid solutions Mg_2_Si_0.4_Sn_0.6_ or Mg_2_Ge_0.4_Sn_0.6_.^[^ ^2c^ ^]^ Moreover we note that for Mg_2_Ge the p‐type properties are better than those of the n‐type while for Mg_2_Si, Mg_2_Sn, and their solid solution the corresponding n‐types are much better. We believe that this is due to the favorable combination of “a” heavy band (HH + LH) and light band (SO) whose energetical differences decrease with temperature.

## 4 Conclusions

We have successfully synthesized Li‐doped Mg_2_Ge using high energy ball milling. The thermolectric properties of Li doped samples exhibit an unusual temperature dependence of *S* and *σ* compared to “standard” highly doped semiconductors. The observed almost constant Seebeck coefficient and pronounced upturn in electrical conductivity at high temperature can be modeled using 2PVB with a temperature dependent interband separation. Here we have taken the heavy hole band and the light hole band as one effective band and the split‐off band as second band; acoustic phonon scattering is taken as the dominant scattering mechanism. The almost constant *S* at high temperature and the upturn in electrical conductivity can be explained by the movement of the SO band with respect to the HH + LH band as temperature increases. While the 2PVB model shows good agreement with the experimental data, we could also rule out by comparative modeling that the observed behavior at high temperature is due to the influence of the minority carriers. The beneficial temperature dependence of Seebeck coefficient and electrical conductivity lead to high *zT* of 0.5 at 700 K for *y* = 0.02 and 0.05 which is superior to p‐Mg_2_Si and Mg_2_Sn and even comparable to the best p‐type solid solutions of Mg_2_Si and Mg_2_Sn. In summary, we show that a non‐rigid band structure with a decreasing interband separation leads to superior thermoelectric properties. This opens a path beyond the standard optimization for further improvement of p‐type Mg_2_(Si, Ge, Sn) solid solutions as well as other materials.

## 5 Experimental Section

Li doped Mg_2_Ge was synthesized via high energy ball milling (SPEX 8000D) using the identical method which was used to prepare p‐type Mg_2_Si_1−_ *_x_*Sn*_x_*.^[^ ^17^ ^]^ The precursors (Mg turnings (Merck), Ge (polycrystalline 99%), and Li granules with purity >99.5%) were weighed according to nominal composition Mg_2_‐yLi_y_Ge. The Li concentration were varied as *y =* 0.01, 0.015, 0.02, and 0.05. The desired elements were transferred into a stainless steel jar with a ball to powder ratio of 1.6:1. All of the experimental steps were conducted inside a glove box under Ar atmosphere to prevent sample oxidation and contamination. The elements were milled with constant speed for 3 h with a halt each hour in between to remove agglomerated powder from the jar walls. We have observed that massive input of mechanical energy (hammering) can lead to powder ignition inside the glove box, so powder removal needs to be done carefully. The obtained fine powders were transferred into a graphite die with a diameter of 13.3 mm and sintered at 923 K for 600 s using a DSP 510 SE from Dr. Fritsch GmbH. The sintering was done under vacuum conditions (≈ 10^−5^ bar) with a sintering pressure of 66 MPa and a heating rate of 1 K s^−1^. The density of the obtained pellets was calculated using Archimedes method.

The obtained pellets were characterized using XRD Siemens D5000 Bragg–Brentano diffractometer with a secondary monochromator, Cu‐K*α* radiations (1.5406 Å) in the range (2*θ*: 20°–80°) and with a step size of 0.01°. The microstructure and phase purity of one of the samples was observed by a EDS detector Zeiss Ultra 55. The temperature dependent electrical conductivity and Seebeck coefficient data was obtained by an in‐house developed four‐probe technique.^[^ ^18^ ^]^ The thermal diffusivity (*α*) was measured by a laser flash technique with a NETZSCH LFA 427 apparatus or with a XFA467HT HyperFlash apparatus. The thermal conductivity (*κ*) was calculated using the relation *κ* = *ρ* *C_p_ α*, where *ρ* and *C_p_* correspond to density of the samples and heat capacity, respectively. The *C_p_* value was obtained from the Dulong–Petit limit $C_{V}^{\mathrm{DP}}:\;C_{p}=C_{V}^{\mathrm{DP}}+\frac{9E_{t}^{2}T}{\beta_{T}\;\rho }$ where *E_t_* ≈ 1.6 × 10^−5^K^[^ ^19^ ^]^ and *β*_*T*_ ≈ 1.7 × 10^−11^Pa, are the coefficient of thermal expansion and an isothermal compressibility, respectively.^[^ ^19^, ^20^ ^]^ In the relevant temperatures *C_p_* increases from 0.632 to 0.649 J g^−1^ K^−1^. The Hall coefficient (*R*_H_) was obtained from Hall measurements using the van der Pauw configuration under a varying magnetic field with maximum value of 0.5 *T*.^[^ ^21^ ^]^ The hall carrier concentration was calculated using the relation $p_{H}=\frac{1}{R_{H}e}$, where *e* is the electronic charge. The uncertainties of the measurements are ± 5%, ±5%, ±8%, and ± 10% for *S*, *σ*, *κ*, and *R*_H_, respectively. The uncertainties are given based on comparison with the NIST standard reference material 3451 and an international round‐robin test.^[^ ^22^ ^]^

## Conflict of Interest

The authors declare no conflict of interest.

## Supporting information

Supporting Information
