# Supplementary material for: Non‐Rigid Band Structure in Mg2Ge for Improved Thermoelectric Performance
Source: Adv Sci (Weinh). 2020 Apr 30;7(12):2000070. doi: 10.1002/advs.202000070 (PMC7312433; doi:10.1002/advs.202000070)
Supplement: Supplementary file 1 — Supporting Information [file ADVS-7-2000070-s001.pdf]

Supporting information

Hasbuna Kamila*, Aryan Sankhla, Mohammad Yasseri, Eckhard Mueller, and Johannes de Boor*

H. Kamila[a], *A. Sankhla*[a], *M. Yasseri*[a,b], *E. Mueller*[a,b], and *J. de Boor*[a]

[a] Institute of Materials Research, German Aerospace Center (DLR), 51147 Koeln, Germany

[b] Institute of Inorganic and Analytical Chemistry, Justus Liebig University Giessen, 35392 Giessen, Germany

E-mail: hasbuna.kamila@dlr.de and Johannes.deboor@dlr.de

1. Microstructure

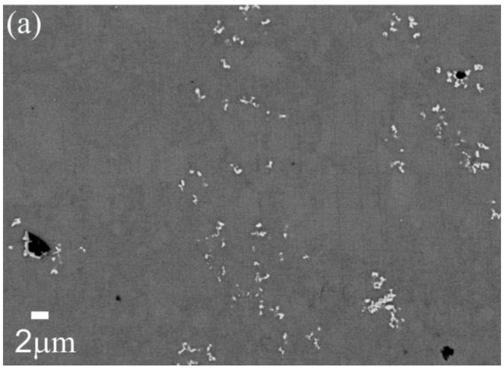

Figure S 1 SEM-BSE image on a large area of $Mg_{1.98}Li_{0.02}Ge$ sample with white particles spreaded unevenly on the sample surface. These white particles are elemental Ge.

2. Thermoelectric properties

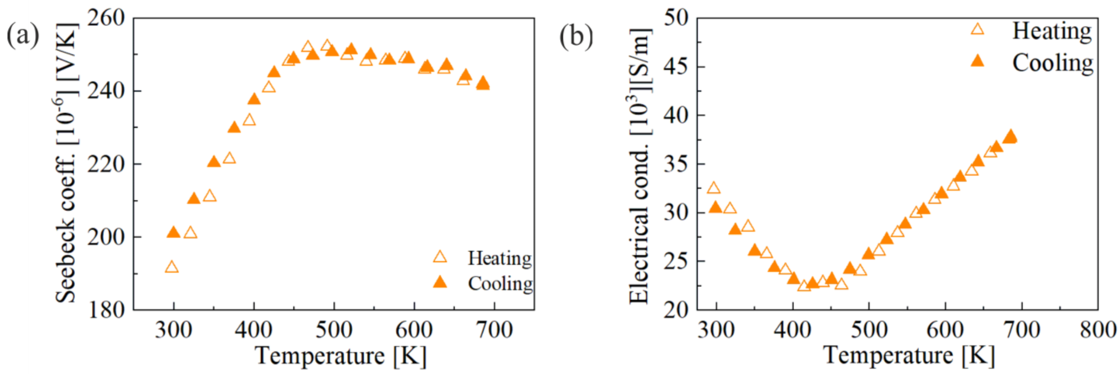

Figure S 2 Heating and cooling data of (a) Seebeck coefficient and (b) electrical conductivity for $Mg_{1.98}Li_{0.02}Ge$. Little discrepancy was observed between the heating and the cooling curves suggesting the sample was stable for the complete measurement cycle.

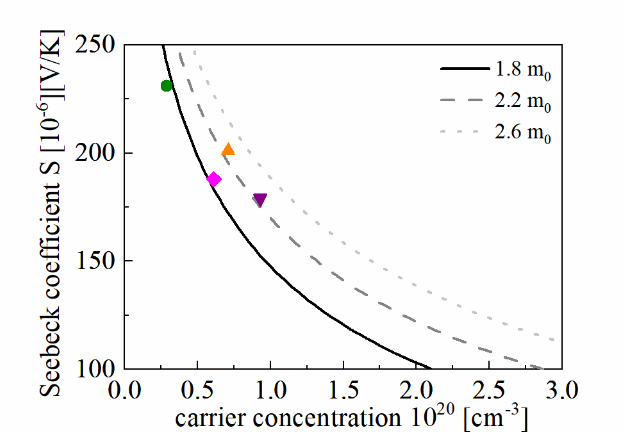

Figure S 3 Seebeck coefficient at room temperature vs carrier concentration (the Pisarenko plot) with $\boldsymbol{m_D^*}$between 1.8 and 2.6 $\boldsymbol{m_0}$ for p-type Mg_2Ge, assuming a single parabolic band model

3. Modelling

a) Model system with onevalence band (VB) and one conduction band (CB) with different temperature dependent band gaps

We model the minority carrier effects by assuming a 1VB and 1CB system, testing two different temperature dependent band gaps. The first case is according to [1] and describes a weak temperature dependence ($E_G = 5.7 - 1.8 \times 10^{-4} T$) of the band gap, see Figure 2a; the other calculation parameters are listed in the Table 1.

Table 1. Input parameters for the modelling of p-type Mg_2Ge using a 1VB+1CB system.

Parameters	VB	CB
Density of states effective mass (m_D^*) [m_0]	2.5	0.49[2]
Valley of degeneracy (N_v)	2[3]	3[4]
Deformation potential (E_{Def})	9[3]	9.8[4]
Elastic constant (C_l) [Pa]	1.17×10^{11} [5]	
Carrier concentration (p) [cm^{-3}]	1.05×10^{20}	

The input parameters for the 1VB+1CB system are listed in the Table 1. The deformation potential between VB and CB is different due to the different position of k point (VBM at Γ point and CBM at X point) [6]. As literature data for Mg_2Ge is very sparse and no value of $E_{Def,CB}$ has been published previously we have used data from Mg_2(Si,Sn), the value ($E_{Def,CB} = 9.8\ eV$ was obtained from the analysis of the samples published in [2] and similar to the value obtained for Mg_2Si [4]. The carrier concentration is assumed to be $p = 1.05 \times 10^{20}\ \mathrm{cm}^{-3}$. The carrier concentration is assumed so that the model fits with the experimental data.

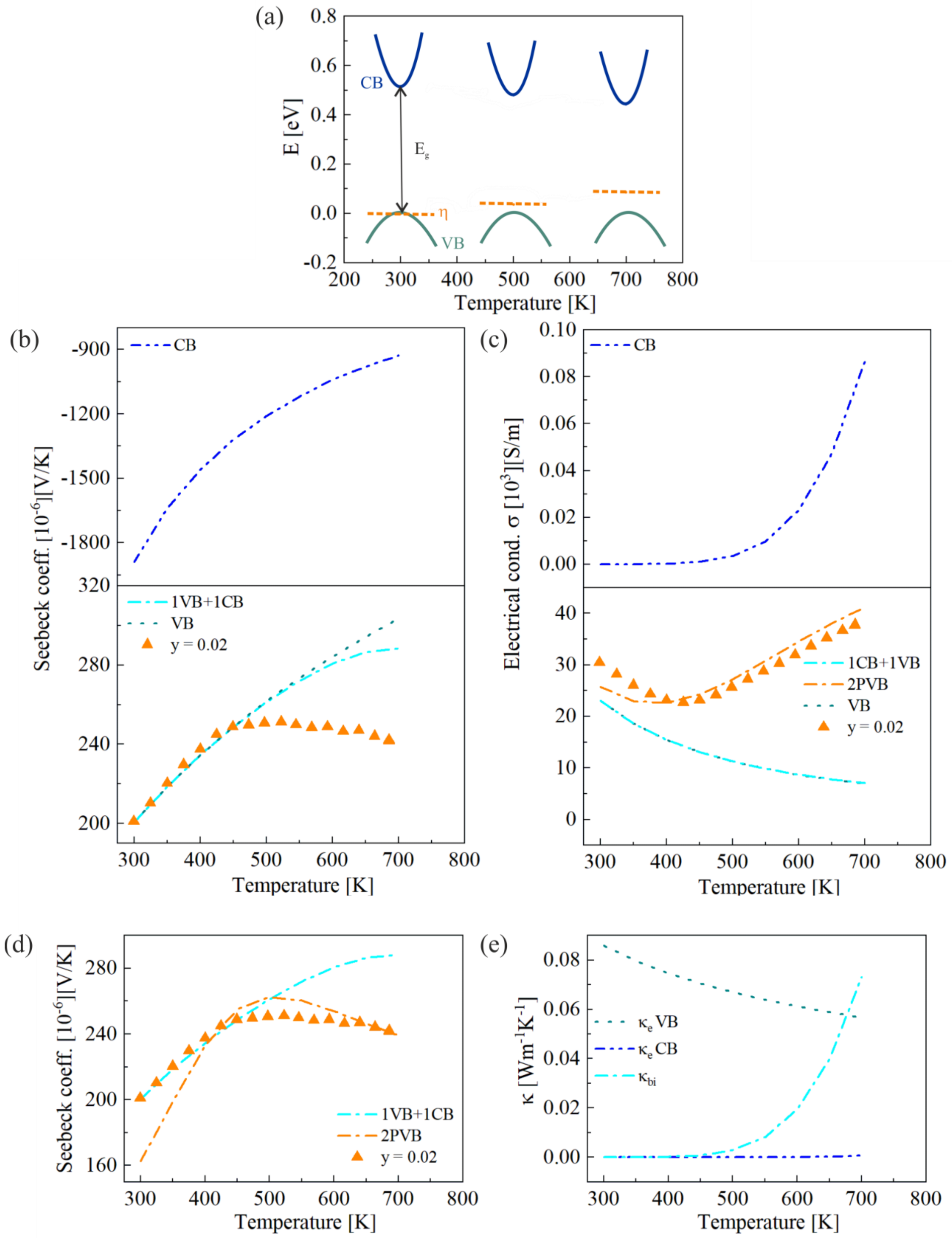

Figure S 4 (a) Schematic of the movement of the conduction band (blue) with respect to the valence band (dark cyan) with temperature dependent band gap ($\boldsymbol{E_G = 0.57 - 1.8 \times 10^{-4} T}$). (b) shows the comparison of the measured *S(T)* with a 1VB+1CB model; with the $\boldsymbol{S}$ of the CB (blue dashed dotted dotted lines), of the VB (dark cyan dotted lines) and the 1VB+1CB (cyan dashed dotted lines) system (c) the comparison of the measured $\sigma(\boldsymbol{T})$ with the model and (d) show the comparison of the measured $\boldsymbol{S(T)}$ with 1VB+1CB system and 2PVB system (orange dashed dotted lines). (e) Temperature dependent κ_e of each band and κ_{bi} are calculated.

As can be seen from Figure S 4b and c, the contribution of the CB to the total Seebeck coefficient and the total electrical conductivity can be neglected. Figure S 4d shows that temperature dependence of Seebeck coefficient for the 1VB+1CB system increases as temperature increases and approaches a maximum beyond 700 K while experimentally we observe a weak maximum at around 500 K, in agreement with the 2PVB model. Also, the electrical conductivity of the 1CB+1VB model does not resemble our thermoelectric properties, σ decreases as temperature increases while in our case, σ decreases till a minimum at 425 K followed by a relatively sharp increase at higher T. We furthermore observe that the bipolar thermal conductivity is small for the chosen parameters.

For the second case with $\boldsymbol{E_G(T) = 0.74 - 8 \times 10^{-4} T}$ according to [7], the decrease in energetical difference between CB and VB is much stronger as temperature increases (Figure S 5a). Figure S 5b and c show that the contribution of CB to the total Seebeck coefficient and electrical conductivity is significant at high temperatures. However, the decrease in S for the 1VB+1CB system is much stronger than shown by the experimental data and the "plateau" region is not observed (Figure S 5). Furthermore, while the predict total electrical conductivity shows an increase at high temperatures it is much later (in temperature) than that of our experimental data (Figure S 5c). Assuming an even smaller band gap or a stronger temperature dependence might lead to a better fit for σ fits better, however would also increase the disparity between model and experiment for $\boldsymbol{S}$ at the same time. We also note that the predicted bipolar thermal conductivity (Equation 7 in the manuscript) becomes comparable to the total thermal conductivity at the highest temperatures in clear disagreement with the experimental observation.

From the discussion of two different 1VB+1CB systems (one with a large band gap at high temperature, the other with a small one) we can deduce that the observed behavior of *S(T)* and *σ(T)* cannnot be explained by 1VB+1CB model and the observed changes are thus notdue to the classical bipolar effect.

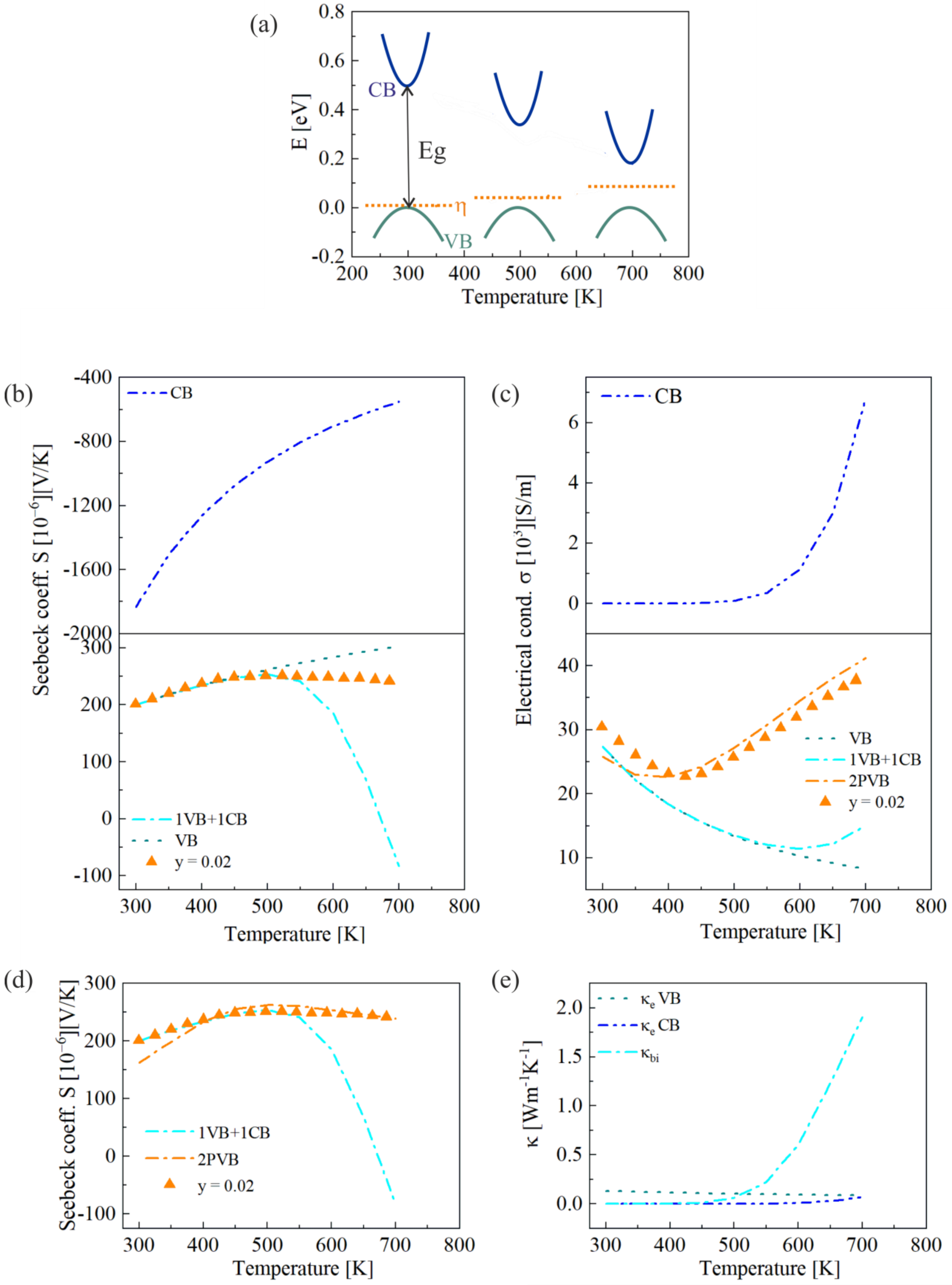

Figure S 5 Schematic of the movement of the conduction band (blue) with respect to the valence band (dark cyan) with temperature dependent band gap ($\boldsymbol{E_G = 0.74 - 8 \times 10^{-4}T}$). (b) shows the comparison of the measured *S(T)* with a 1VB+1CB model; with the $\boldsymbol{S}$ of the CB (blue dashed dotted dotted lines), of the VB (dark cyan dotted lines) and the 1VB+1CB (cyan dashed dotted lines) system (c) the comparison of the measured $\sigma(\boldsymbol{T})$ with the model and (d) show the comparison of the measured $\boldsymbol{S(T)}$ with 1VB+1CB system and 2PVB system (orange dashed dotted lines). (e) Temperature dependent κ_e of each band and κ_{bi} are calculated.

The obtained result for κ_{bi} can be used to estimate the temperature dependence of the band gap by trying to match the calculated κ_{bi} with the experimental.

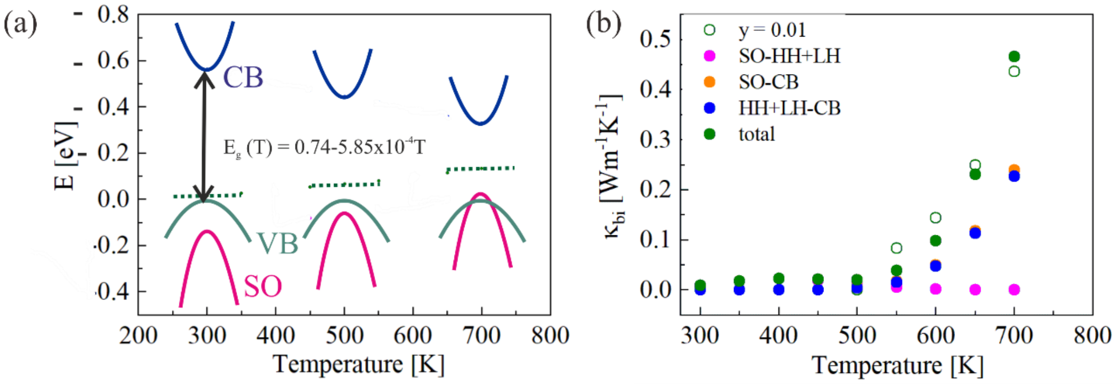

Figure S 6 (a) Schematic of the movement of the conduction band (blue) and the SO band (magenta) with respect to the HH+LH band (dark cyan) with respect to temperature dependent band gap ($\boldsymbol{E_G = 0.74 - 5.85 \times 10^{-4} T}$). (b) Temperature dependent κ_{bi} of each band are calculated for $Mg_{2-y}Li_yGe$ with $\boldsymbol{y = 0.01}$ (empty symbols). As we are considering here three bands there are also three bipolar contributions: that for carrier pairs from SO and the effective HH+LH band, for carrier pairs from SO and CB and from HH+LH and CB. The contribution from SO and HH+LH is small as both carriers have the same sign.

For $E_g(T) = 0.74 - 5.85 \times 10^{-4} T$ we find agreement between the calculated and the measured bipolar thermal conductivity (see Figure 5b). This is somewhat in between the previously considered cases from literature $E_g(T) = 0.57 - 1.8 \times 10^{-4} T$ [1] and $E_g(T) = 0.74 - 8 \times 10^{-4} T$ [7] and justifies that the conclusions we obtained from the analysis of these cases are generally valid for the material system.

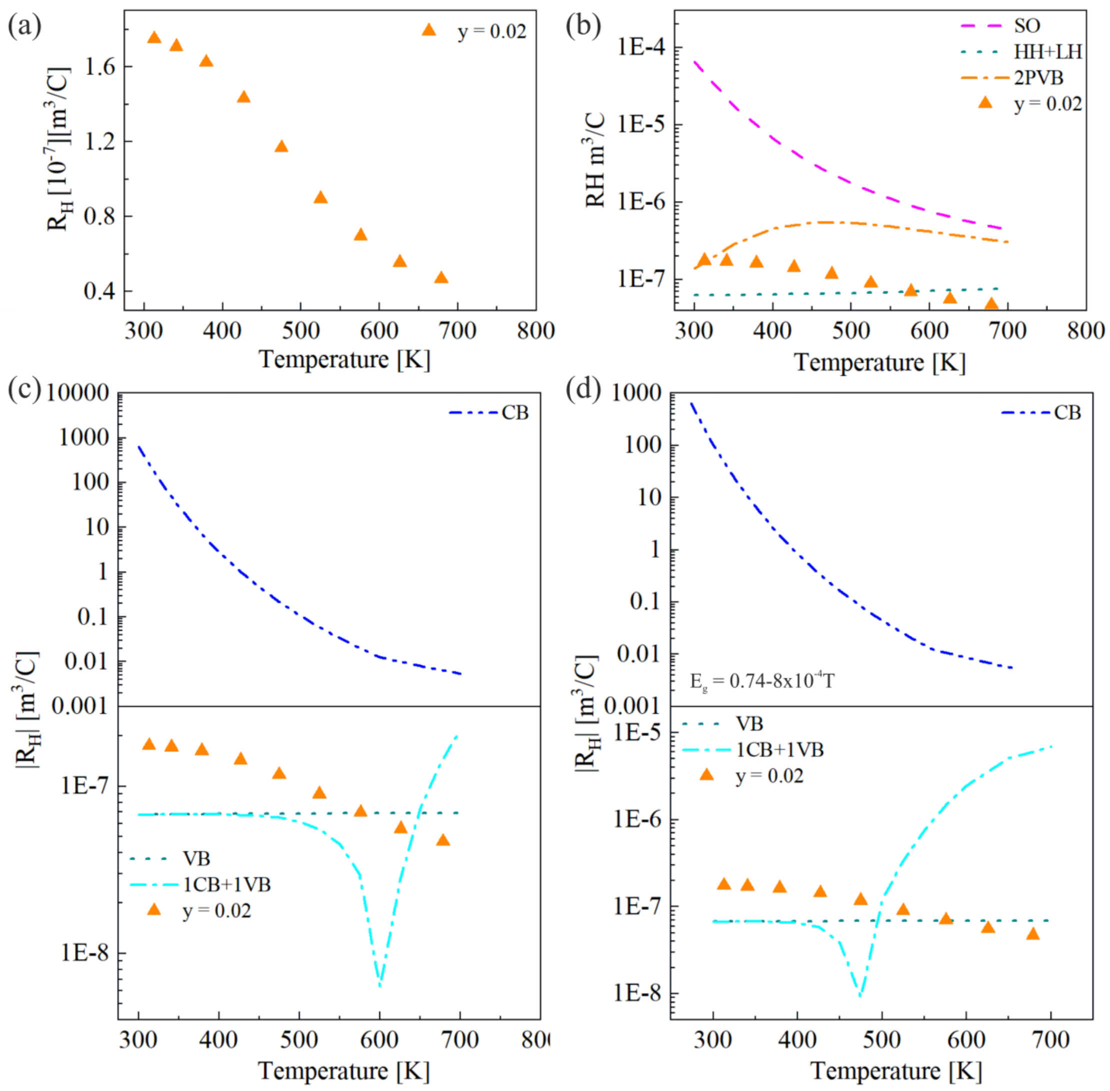

Figure S 7 Temperature dependence of the measured (a) Hall coefficient (R_H) for $Mg_{1.98}Li_{0.02}Ge$ with y = 0.02. (b) shows the comparison of the measured R_H(T) with the 2PVB model; with the R_H of the SO band (magenta dashed lines), of the HH+LH band (dark cyan dotted lines) and the total 2PVB system (orange dashed dotted lines). (c) and (d) show the comparison with the 1VB+1CB model with the contribution of the CB (blue dashed dotted dotted lines), the VB (dark cyan dotted lines) and 1VB+1CB system (cyan dashed dotted lines). (c) shows the comparison with a relatively large band gap at high T E_G (T) $=$0.57-1.8× $10^{-4}T$ and (d) that of a smaller band gap at high temperature: E_G (T) =0.74-8× $10^{-4}T$.

The temperature dependence of Hall coefficient (R_H) is shown in Figure 1(a) and is taken from the cooling data. Figure 1a shows a strong temperature dependent of R_H which is decreasing with increasing temperature. This is a clear indication for the influence of (at least) a second band, either the conduction band or further valence band [8]. The Hall coefficients for the 1CB+1VB and the 2PVB system are calculated according to the equations from the manuscript and the parameters from Table 1.

Comparing the experimental result with the 2PVB system we do not find total agreement (Figure 1b). The agreement is good at low temperatures, but the temperature dependence of R_H is

different between 300 K and 450 K. At higher temperatures both the prediction and the experimental result show a visible, but relatively weak temperature dependence. Quantitatively the values agree within a factor of 10 over the whole temperature range.

Comparison with the 1VB+1CB system (c and d), however, shows a much stronger disagreement. For both assumed descriptions of the band gap, the Hall coefficient shows a change of sign and a very strong change with temperature in the neighboring temperature ranges, both features are not observed experimentally. The change of sign arises from the higher mobility of the electrons compared to those of the holes (see Equation 8), which is a general feature of the Mg_2X system [4]. The zero crossing for the Hall coefficient is thus a universal feature for a p-Mg_2X system where the minority carriers start to become relevant and not due to our choice of parameters. Note also that the difference between experimental value and prediction is partially several orders of magnitude for the 1VB+1CB system. We thus deduce that the observed temperature dependence of the Hall coefficient cannot be explained by the influence of the minority carriers.

For a two band system with a convergence of the two relevant bands, R_H is predicted to possibly show a maximum value and a visible, but relatively weak temperature dependence [10]. Note however, that the maximum is not a universal feature and note also that the temperature where it supposed to occur, depends on mobility and carrier concentration ratios [11]. While for the 2PVB system we predict such a maximum (at around 450 K), we do not see this in the experimental data, which decreases monotonously with temperature. However, we note that $\frac{\partial R_H}{\partial T}$ is close to zero around room temperature, possibly indicating a maximum just below room temperature.

We believe the fact that we do not get a prefect agreement between the experimental result and the prediction from the 2PVB system is the simplified description of the system. P-type Mg_2X is known to have three valence bands, two of which we treat as one effective band. While this does not affect the modelling of the Seebeck coefficient, it matters for the conductivities and the Hall coefficients, generally the Hall coefficient of a system with two distinct bands is not the same as that of a system with two identical bands. Modelling a 3PVB system would certainly cause a better fit for R_H, however, we do not have sufficient experimental input to avoid a heavily underdefined system, a better agreement because ofmany adjustable parameters would therefore not carry a lot of meaning.

b) A 2PVB model Without Interband separation (IBS)

We have assumed the HH and the LH bands are degenerate and systematically change the effective mass and carrier concentration such that the model fits with the experimental data. However, the modeled data does not fit well with our data (Figure S8 and S9).

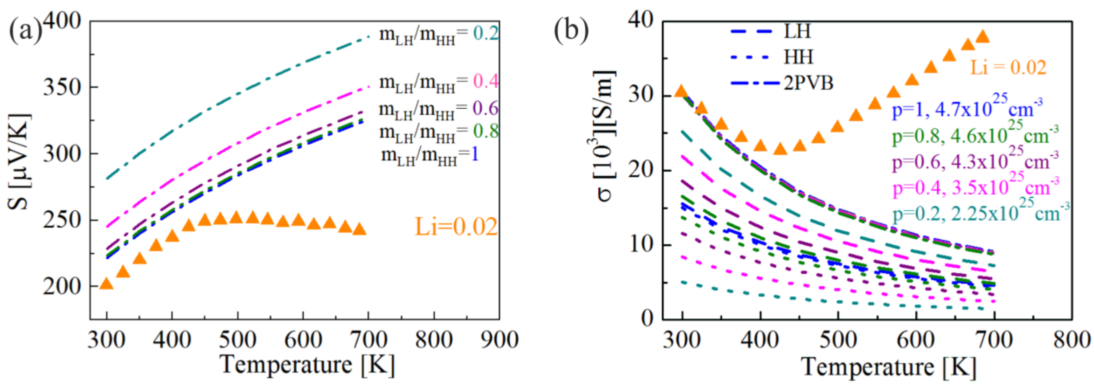

Figure S 8 Temperature dependence of (a) Seebeck coefficient and (b) electrical conductivity experimental data is fitted by tuning the effective mass ratio ($\frac{m_{LH}}{m_{HH}}$ = 0, 0.2, 0.4, 0.6, 0.8, and 1) and roughly adjusting the carrier concentration so that the model fits with the experimental electrical conductivity at least at room temperature. The modeled data does not show the temperature dependence as observed in the experimental data for our sample.

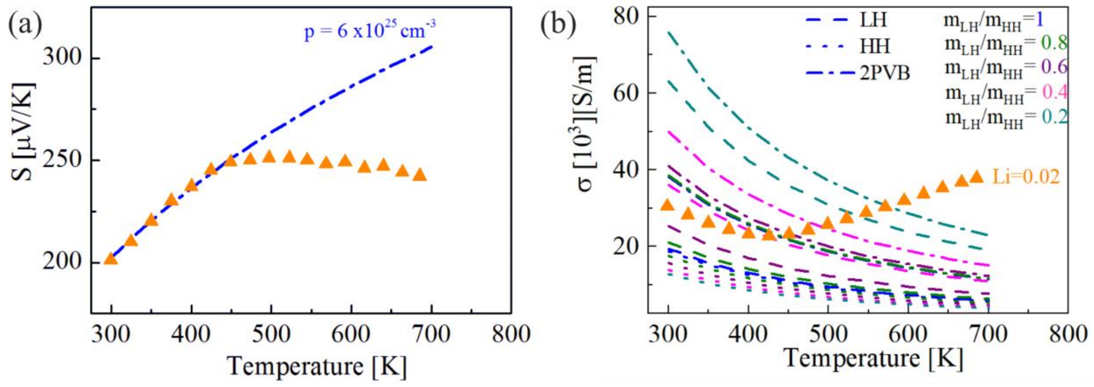

Figure S 9 Temperature dependence of (a) Seebeck coefficient and (b) electrical conductivity. The experimental data is fitted by tuning the effective mass ratio ($\frac{m_{LH}}{m_{HH}}$ = 0, 0.2, 0.4, 0.6, 0.8, and 1) and constant carrier concentration so that the model fits with the experimental data for *S* at least at room temperature. The modeled Seebeck coefficient increases as temperature increases (> 450 K) which is inconsistent with our experimental data.

c) A 2PVB model with Interband separation (IBS)

In principle, we have an under defined system with some degree of freedom with the choice of parameters. Thus, we tested different parameters which mostly lead to a poor fit. However, this also implies that the absolute values of the model parameters are subject to significant uncertainty.

The Figures 10 and S11 show that the model reproduces the experimental data only with the right temperature dependent interband separation and lower m_{SO}.

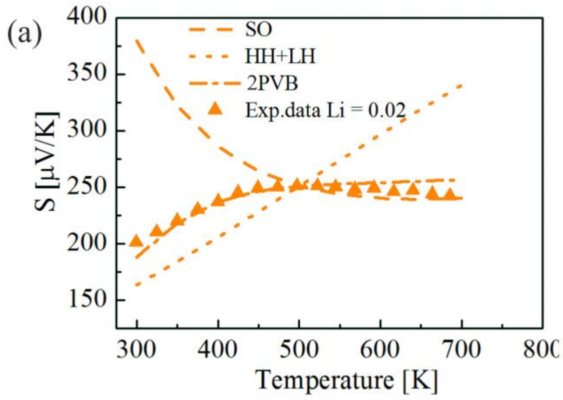

Figure S 10 Temperature dependent modeled and experimental (a) Seebeck coefficient and (b) electrical conductivity data of 2% Li-doped Mg_2Ge.The experimental data is fitted with ΔE = (-0.2+4×10^{-4}T)e/$\boldsymbol{k_B}$T and higher SO effective mass ($\boldsymbol{m_{SO} = 1.0\, m_0}$, $\boldsymbol{m_D^* = 2.1\, m_0}$). The model reproduces the experimental Seebeck coefficient data very well however it is not the case for the electrical conductivity.

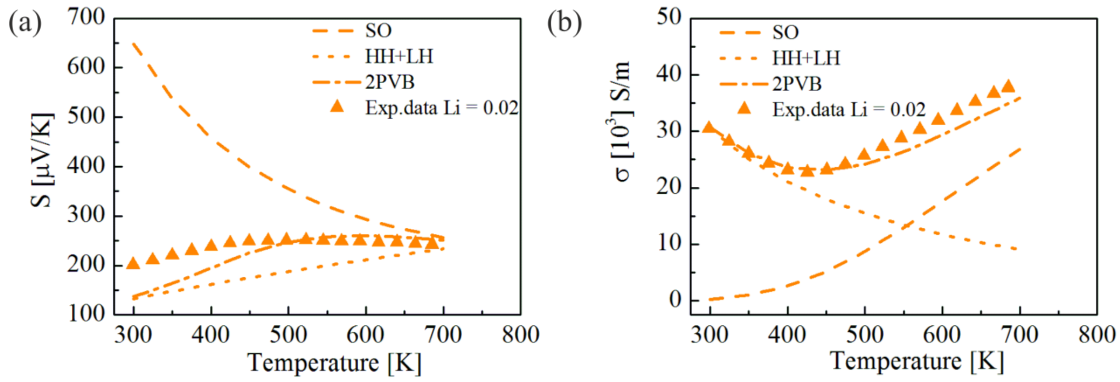

Figure S 11 Temperature dependent modeled and experimental (a) Seebeck coefficient and (b) electrical conductivity data of 2% Li-doped Mg_2Ge. The experimental data is fitted with ΔE = (-0.3+4×10^{-4}T)e/$\boldsymbol{k_B}$T and lower SO effective mass ($\boldsymbol{m_{SO} = 0.5\, m_0, m_D^* = 2.1\, m_0}$). The model reproduces both the *S* and σ experimental data qualitatively well however, there is large discrepancy between the modeled and the experimental *S(T)* data between 300 and 500 K.

Figure S12-S13 show the temperature dependent of transport data to be quite sensitive to ΔE.

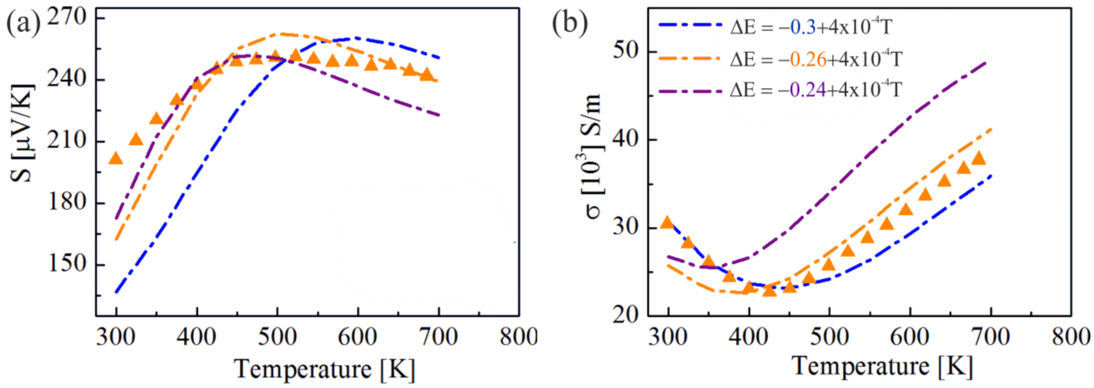

Figure S 12 Temperature dependent modeled and experimental (a) Seebeck coefficient and (b) electrical conductivity data of 2% Li-doped Mg_2Ge. The experimental data is fitted with different $\Delta E = (-x+\mathbf{4}\times10^{-4}T)e/\boldsymbol{k_B}T$ (where x = 0.24, 0.26, and 0.3) and constant SO effective mass ($\boldsymbol{m_{SO}} = \mathbf{0.5}\,\boldsymbol{m_0}$, $\boldsymbol{m_D^*} = \mathbf{2.1}\,\boldsymbol{m_0}$). The temperature dependence of the calculated $\boldsymbol{S}$ and $\boldsymbol{\sigma}$ are quite sensitive to ΔE thus the behavior of temperature dependent transport data changes with changing in ΔE values.

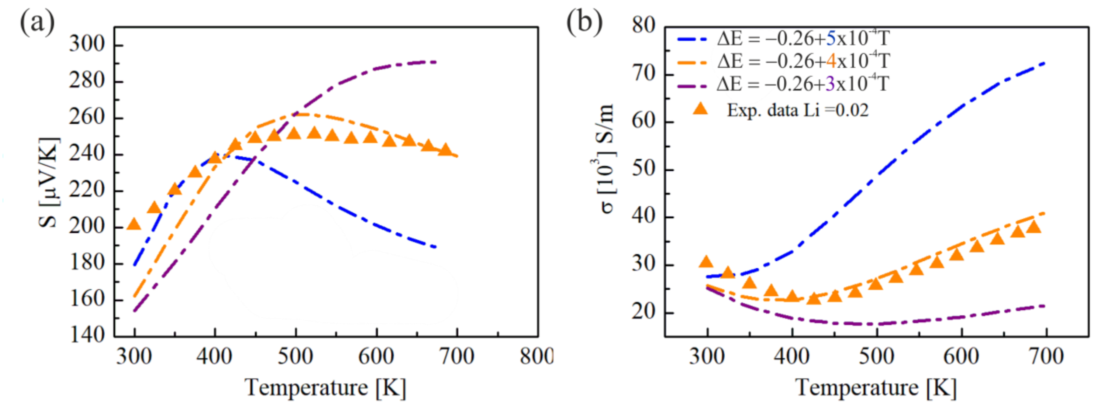

Figure S 13 Temperature dependent modeled and experimental (a) Seebeck coefficient and (b) electrical conductivity of 2% Li-doped Mg_2Ge samples. The experimental data is fitted with different temperature dependent, $\Delta E = (-0.26+\boldsymbol{x}\times10^{-4}T)e/\boldsymbol{k_B}T$ (where x = 3, 4, and 5) and constant SO effective mass ($\boldsymbol{m_{SO}} = \mathbf{0.5}\,\boldsymbol{m_0}$, $\boldsymbol{m_D^*} = \mathbf{2.1}\,\boldsymbol{m_0}$). The temperature dependent modeled Seebeck coefficient and electrical conductivity was observed to be quite sensitive to the inter-band separation energy (ΔE).

Figure S14 shows that the effect of the second band is negligible for extremely larger inter-band separation.

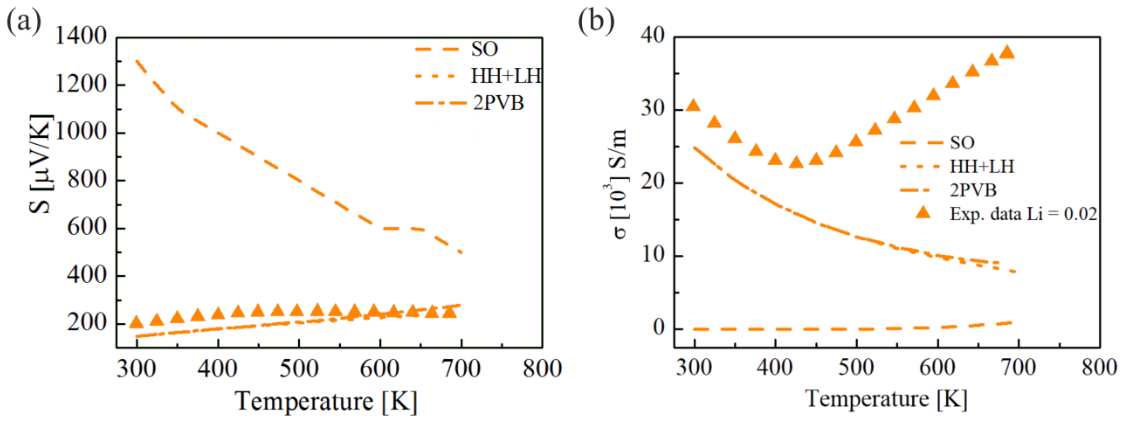

Figure S 14 Temperature dependent modeled and experimental (a) Seebeck coefficient and (b) electrical conductivity data of 2% Li-doped Mg_2Ge. The experimental data is fitted with different ΔE = (-0.5+4×10^{-4}T)e/$\boldsymbol{k_B}$T and constant SO effective mass ($\boldsymbol{m_{SO} = 0.5\, m_0}$). The effect of SO band is basically negligible due to large ΔE compared to our value thus the S and σ model is mainly governed by the HH+LH band.

We have also tried to fit the experimental data of the samples with different Li concentration with higher SO effective mass. The model reproduces the electrical conductivity very well however there lies a discrepancy in the modeled and experimental data of the Seebeck coefficient.

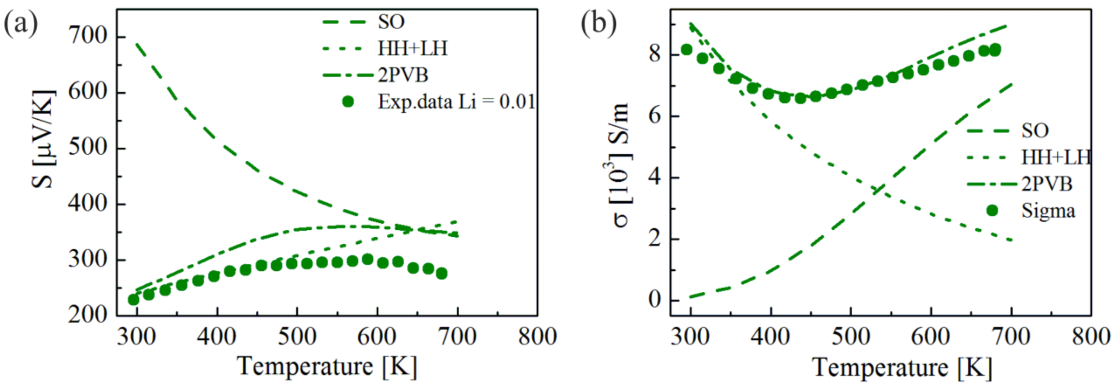

Figure S 15 Temperature dependent modeled and experimental (a) Seebeck coefficient and (b) electrical conductivity data of 1% Li-doped Mg_2Ge. The experimental data is fitted with constant ΔE = (-0.26+4×10^{-4}T)e/$\boldsymbol{k_B}$T and higher SO effective mass ($\boldsymbol{m_{SO} = 0.7\, m_0}$). There is an improvement in the agreement of the modeled data to the experimental σ data.

We have also tried to fit the lighter SO effective mass band ($\frac{m_{HH+LH}}{m_{SO}} = 6$) with the constant interband separation (ΔE = (-0.26+4×10^{-4}T)e/k_BT. However, the result is inconsistence with our experimental data.

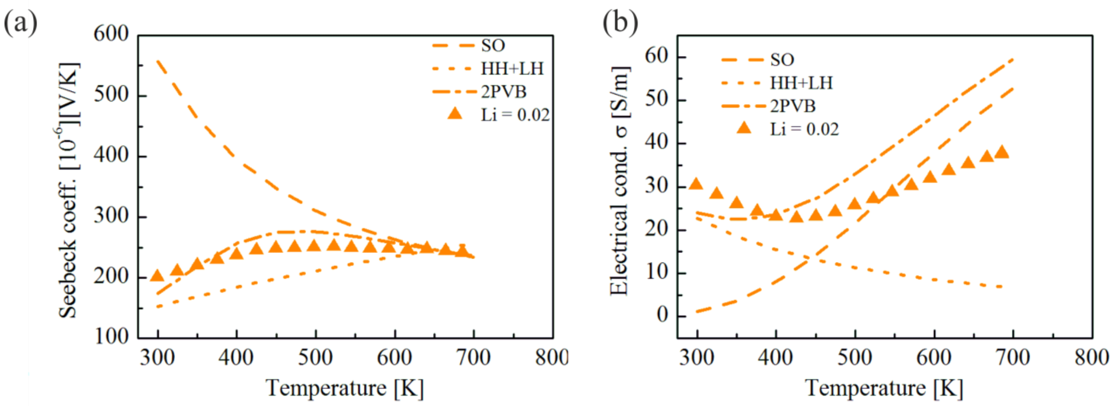

Figure S 16 temperature dependent modeled and experimental (a) Seebeck coefficient and (b) electrical conductivity data of 2% Li-doped Mg_2Ge.The experimental data is fitted with lower SO effective mass ($\frac{\boldsymbol{m_{HH+LH}}}{\boldsymbol{m_{SO}}} \approx$ **6**; $\boldsymbol{m_{SO} = 0.3\, m_0}$), the ΔE = (-0.26+4×$10^{-4}$T)e/$\boldsymbol{k_B}$ and $\boldsymbol{p = 1.1 \times 10^{20} cm^{-3}}$. The temperature dependence of modeled electrical conductivity increases sharply at lower temperatures which is inconsistence with our experimental data.

We have also attempted to model the electrical transport properties by keeping the mass ratio constant with different m_D^* and adjust the carrier concentration such that the model fits with the experimental data. Furthermore, we have employed the lower deformation potential ($E_{Def} = 7.5\, eV$) and using the measured carrier concentration. Both of the models fit with the experimental data however the fits are not getting better (Figure S17-S18).

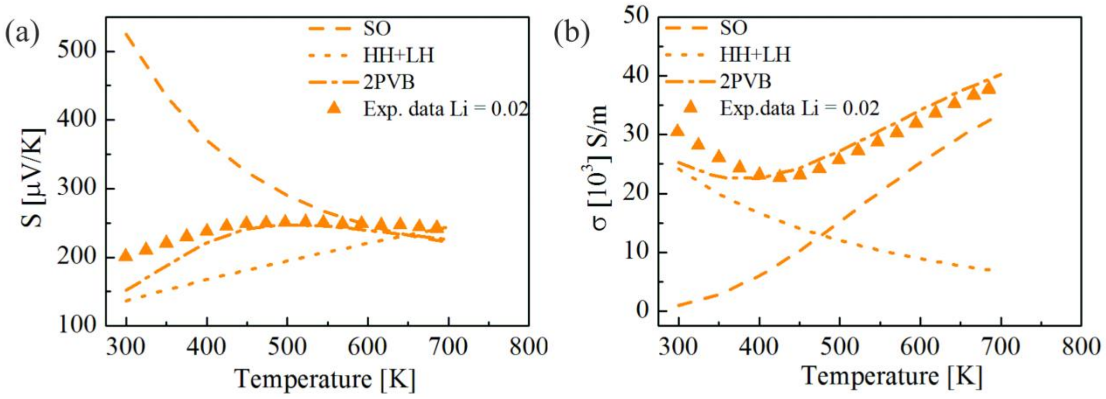

Figure S 17 Temperature dependent modeled and experimental a) Seebeck coefficient and b) electrical conductivity data of 2% Li-doped Mg_2Ge.The experimental data is fitted by keeping the mass ratio constant ($\frac{\boldsymbol{m_{HH+LH}}}{\boldsymbol{m_{SO}}} \approx \frac{\boldsymbol{2.3\, m_0}}{\boldsymbol{0.6\, m_0}} \approx$ **3.8**), ΔE= (-0.26+4×10^{-4}T)e/$\boldsymbol{k_B}$ and $\boldsymbol{p = 1.7 \times 10^{20} cm^{-3}}$. The model qualitatively represents the experimental data, and thus keeping constant the ratio mass and IBS are the important parameters to reproduce the experimental data.

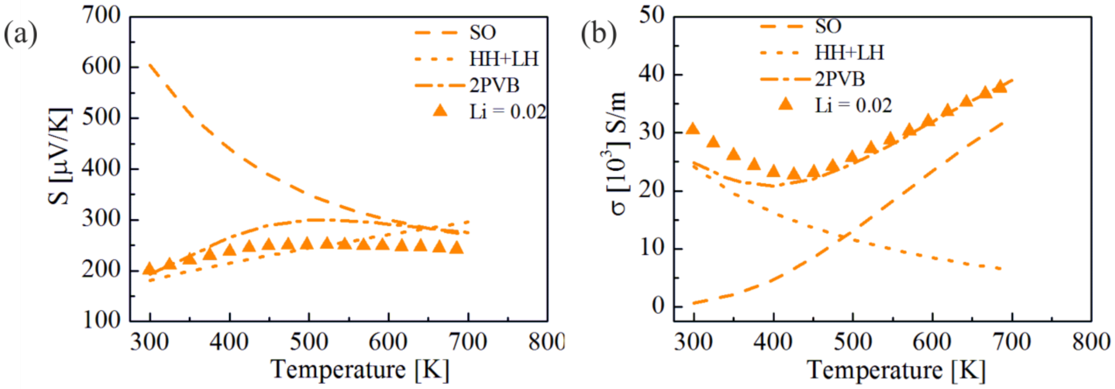

Figure S 18 Temperature dependent modeled and experimental a) Seebeck coefficient and b) electrical conductivity data of 2% Li-doped Mg_2Ge. The experimental data is fitted with lower deformation potential $\boldsymbol{E_{Def} = 7.5\ eV}$ and the obtained carrier concentration (p = 0.7× $\boldsymbol{10^{20}\ cm^{-3}}$). The model reproduces the experimental data qualitatively well however the fit does not get better.

References:

[1] L. A. Lott, D. W. Lynch, *Physical Review* **1966**, 141, 681.

[2] R. Santos, M. Nancarrow, S. X. Dou, S. Aminorroaya Yamini, *Scientific Reports* **2017**, 7, 3988.

[3] H. Kamila, P. Sahu, A. Sankhla, M. Yasseri, H.-N. Pham, T. Dasgupta, E. Mueller, J. de Boor, *Journal of Materials Chemistry A* **2019**, 7, 1045.

[4] D. A. Pshenai-Severin, M. I. Fedorov, A. Y. Samunin, *Journal of Electronic Materials* **2013**, 42, 1707.

[5] J.-i. Tani, M. Takahashi, H. Kido, *Journal of alloys and compounds* **2009**, 485, 764.

[6] K. Kutorasinski, B. Wiendlocha, J. Tobola, S. Kaprzyk, *Physical Review B* **2014**, 89, 115205.

[7] V. K. Zaitsev, M. Fedorov, E. A. Gurieva, I. S. Eremin, P. P. Konstantinov, A. Samunin, M. V. Vedernikov, *Thermoelectrics of n-type with ZT > 1 based on Mg2Si-Mg2Sn solid solutions*, **2005**.

[8] W. Li, S. Lin, X. Zhang, Z. Chen, X. Xu, Y. Pei, *Chemistry of Materials* **2016**, 28, 6227.
